# Therapy de‐escalation for testicular cancer (THERATEST): A multi‐centre observational cohort feasibility study of de‐escalation therapies for good prognosis stage II germ cell tumours

**DOI:** 10.1002/bco2.70057

**Published:** 2025-07-29

**Authors:** Nasreen Abdul Aziz, Kenrick Ng, Constantine Alifrangis, Ben Tran, Ciara Conduit, Elizabeth Liow, Charlotte Ackerman, Ramona Georgescu, Tanim Jamal, Clare Relton, Erik Mayer, David Nicol, Walter Cazzaniga, Robert Huddart, Alison Reid, Jonathan Shamash, Prabhakar Rajan

**Affiliations:** ^1^ Department of Medical Oncology St Bartholomew's Hospital, Barts Health NHS Trust London UK; ^2^ Centre for Experimental Cancer Medicine Barts Cancer Institute, Queen Mary University of London London UK; ^3^ School of Medicine School of Postgraduate Studies, Royal College of Surgeons in Ireland Dublin Ireland; ^4^ Department of Medical Oncology University College London Hospitals NHS Foundation Trust London UK; ^5^ National Institute for Health Research University College London Hospitals Biomedical Research Centre University College London Hospitals NHS Foundation Trust London UK; ^6^ Department of Medical Oncology Peter MacCallum Cancer Centre Melbourne Australia; ^7^ Division of Personalised Oncology Walter and Eliza Hall Institute of Medical Research Parkville Australia; ^8^ Department of Medical Oncology Royal Hobart Hospital Hobart Australia; ^9^ Wolfson Institute of Population Health Queen Mary University of London London UK; ^10^ Department of Urology The Royal Marsden Hospital NHS Foundation Trust London UK; ^11^ Department of Medical Oncology The Royal Marsden Hospital NHS Foundation Trust London UK; ^12^ Centre for Cancer Cell and Molecular Biology Barts Cancer Institute, Queen Mary University of London London UK; ^13^ Department of Urology The Royal London Hospital, Barts Health NHS Trust London UK; ^14^ Department of Urology University College London Hospitals NHS Foundation Trust London UK

**Keywords:** carboplatin AUC10, De‐escalation therapy, feasibility study, pragmatic study, rRPLND, stage II seminoma

## Abstract

**Background:**

Standard of care (SOC) treatments for International Germ Cell Cancer Collaborative Group (IGCCCG) good prognosis stage II germ cell tumours (GCT) involve primary orchidectomy followed by combination chemotherapy for both seminoma and non‐seminomatous germ cell tumours (NSGCT). Alternatively, external beam radiotherapy may be used for seminoma and retroperitoneal lymph node dissection (RPLND) for NSGCT. While these treatments achieve high cure rates, they are associated with significant toxicities. De‐escalation strategies including three cycles of Carboplatin AUC10 or robotic RPLND with or without adjuvant chemotherapy have demonstrated potential to reduce treatment‐related toxicity in stage II seminoma while preserving oncological efficacy. However, these approaches are not widely adopted due to limited prospective comparative trials.

**Study Design:**

The THERATEST trial is a prospective multicentre observational feasibility study evaluating participants receiving SOC treatments for good prognosis stage II seminoma and NSGCT or de‐escalated treatments for stage II seminoma.

**Endpoints:**

The primary endpoints are to assess feasibility of recruitment and retention. Secondary endpoints include assessing health‐related quality of life (HRQOL), sexual function and satisfaction, progression‐free survival (PFS), overall survival (OS) and safety and treatment‐related complications.

**Patients and Methods:**

Thirty participants with good prognosis stage II seminoma or NSGCTs will be recruited over 18 months into two cohorts: de‐escalation arm and SOC arm. The de‐escalation cohort will receive either Carboplatin AUC10 or robotic RPLND with or without adjuvant therapy depending on institutional SOC. Participants who decline or are ineligible for de‐escalation will receive SOC treatment: combination chemotherapy or radiotherapy for seminoma and combination chemotherapy for NSGCT. All participants will be followed for two years post‐treatment or until withdrawal. Data collection includes recruitment and retention rates, disease status, surgical outcomes, adverse events and patient‐reported outcomes using validated questionnaire: EORTC QLQ‐TC26, EORTC QLQ‐C30, Brief Male Sexual Function Inventory (BMSFI) and additional enquiries on anejaculation.

**Coordinating Centre:**

THERATEST Trial Coordinator, Centre for Experimental Cancer Medicine, Barts Cancer Institute, Queen Mary University of London, Old Anatomy Building, Charterhouse Square, London, EC1M 6BQ|T: 0207882 8497|E: bci-theratest@qmul.ac.uk

**Trial registration number:**

ISRCTN61007118.

## 1 BACKGROUND

Standard of care (SOC) treatments for International Germ Cell Cancer Collaborative Group (IGCCCG) good prognosis metastatic testicular germ cell tumours (TGCT) include primary orchidectomy followed by combination chemotherapy (three cycles of Bleomycin, Etoposide and Cisplatin (BEP) or four cycles of Etoposide and Cisplatin (EP))^1^ with cure rates exceeding 95%.^2^ Alternatives are external beam radiotherapy (30–36 Gy in 2Gy fractions) for stage IIA/B seminoma, and primary retroperitoneal lymph node dissection (RPLND) for selected marker‐negative stage IIA/IIB non‐seminomatous germ cell tumour (NSGCT). However, combination chemotherapy is associated with early haematological and non‐haematological toxicities (reviewed in^3^) and radiotherapy can lead to late effects, including second malignancies, resulting in reduced utilisation in some centres.^4^ RPLND is typically an open surgical procedure with associated morbidity, and key long‐term side effect is ejaculatory dysfunction due to surgical damage to the lumbar sympathetic chain and pre‐aortic plexus.

Within the good prognosis group, stage II seminoma shows the most favourable oncological outcomes with long‐term cure rates of exceeding 98%,^3^ leading to explorations of non‐SOC therapy de‐escalation to reduce treatment‐related toxicity and maintain oncological outcomes.^3^ A prospective multicentre study of a single cycle of neoadjuvant Carboplatin AUC7 with reduced field and dose of radiotherapy for stage IIA/B seminoma yielded 3‐year progression‐free survival (PFS) rate of 93.7% with minimal toxicity.^5^ In a multi‐centre cohort, three cycles of Carboplatin AUC10 monotherapy for stage II seminoma yielded a 2‐year PFS rate of 96.5% and a 3‐year cancer‐specific survival (CSS) rate of 100%^6^ with only myelosuppression being a major acute toxicity. A recent prospective clinical trial of RPLND without adjuvant chemotherapy in stage IIA/B seminoma showed a 31% recurrence rate over a mean of 26 months follow‐up.^7^ Another prospective trial of RPLND reported an 81% 2‐year recurrence free survival (RFS) and low toxicity in small volume stage IIA/B disease.^8^ Similarly, a prospective trial of nerve‐sparing RPLND for stage IIA/B seminoma showed a 10% recurrence rate at up to 9 months follow‐up.^9^ To reduce the recurrence risk, one group has adopted a single cycle of adjuvant Carboplatin AUC7 after minimally‐invasive robot‐assisted RPLND (rRPLND), which appears to have low toxicity and relapse rates.^10^

Despite high PFS rates, fewer toxicities and better quality of life outcomes, de‐escalated treatments are not yet widely adopted as SOC primarily due to a lack of comparative outcomes data derived from prospective randomised clinical trials and limited adoption of minimally invasive RPLND. Consequently, a recent UK survey highlighted significant geographical heterogeneity in SOC treatments for stage II seminoma, and only a third of centres have adopted a treatment de‐escalation regimen.^11^ TGCTs are a rare cancer and account for only approximately 1% of newly diagnosed malignant tumours,^12^ hence there are challenges in recruitment to randomised controlled trials (RCTs), including ethical concerns over randomisation without clear equipoise, and patient and clinician biases regarding SOC and de‐escalated options.

THERApy de‐escalation in TESTicular cancer (THERATEST) is a multi‐centre observational cohort feasibility study of patients receiving guideline‐recommended SOC treatments for good prognosis stage II seminoma (combination chemotherapy or radiotherapy) and NSGCT (combination chemotherapy) or novel de‐escalated treatments (primary rRPLND or Carboplatin AUC10) for stage II seminoma. In view of the rarity of testicular cancer, THERATEST adopts a pragmatic cohort design aimed at maximising participants' recruitment and reducing attrition, while aligning treatment strategies with institutional SOC. The study findings will inform the design and conduct of a future definitive trial, which may adopt a pragmatic cohort‐embedded design, by providing prospectively‐collected information on the safety and complications of treatments as well as health‐related quality of life (HRQOL) outcomes.

## 2 STUDY DESIGN

The THERATEST trial is a multicentre observational feasibility of two cohort study. It includes patients receiving SOC treatments for good prognosis stage II seminoma (combination chemotherapy or radiotherapy) and NSGCT (combination chemotherapy) or de‐escalated treatments (primary rRPLND or Carboplatin AUC10) for stage II seminoma. If participants are not eligible for either of these de‐escalation strategies or decline treatment, they will be offered either combination chemotherapy or radiotherapy but remain within the study. In both cohorts, participants will be followed up for 2 years after treatment completion. Beyond the study period, participants will be followed up as per institutional SOC protocols as part of prospective institutional audits.

### 2.1 Study settings

Three recruiting sites are planned with additional sites being added via a future protocol amendment. Participants will be recruited from within the National Health Service (NHS).

### 2.2 Recruitment

Thirty evaluable participants with histologically confirmed good prognosis stage II seminoma and NSGCT who have not previously received chemotherapy or radiotherapy will be enrolled from UK sites. A minimum of 5 participants will be recruited into each treatment de‐escalation group within each cohort, and a maximum of 15 patients will be recruited into the SOC groups. In the event of withdrawal, participants will not be replaced in this feasibility study. Participants will be identified in the secondary care setting via supra‐regional multi‐disciplinary team (MDT) meetings at research sites.

## 3 END POINTS

### 3.1 Primary objective and endpoint

The primary objective of this study is to assess the feasibility of recruitment and retention by measuring the number of participants recruited per month and retained annually. The primary endpoint for this study is the successful recruitment of 30 participants into the THERATEST study within 18 months from the first participant enrolled.

### 3.2 Secondary objectives and endpoints

1 Health‐Related Quality of Life (HRQOL)

2 Sexual Drive, Function and Satisfaction

3 Progression‐Free Survival (PFS) and Overall Survival (OS)

- PFS at 2 years is defined as the proportion of participants who do not experience disease progression or death from any cause within the 2‐year follow up period.
- OS at 2 years is defined as the proportion of participants who remain alive from any cause at the 2‐year follow up period.
  The study aims for these survival rates to remain consistent with SOC treatments (>95%).4. Safety and Treatment‐Related Complications

4 Safety and Treatment‐Related Complications

- The incidence, nature and severity of adverse events (AEs) will be documented according to the Common Terminology Criteria for Adverse Events (CTCAE) v5.0, from the time of informed consent until 6 weeks post‐surgery or 6 months post‐chemotherapy.
- Surgical complications will be classified using the Clavien‐Dindo scoring system, and additional safety assessments will include analysis of blood transfusion rates, ITU (Intensive Therapy Unit) admissions and dialysis requirements from patient records up to 6 months post‐rRPLND.

The study aims to evaluate changes in HRQOL before and after treatment(s). This will be assessed by measuring changes in domain scale scores and single‐item scores from the European Organisation for Research and Treatment of Cancer (EORTC) QLQ‐TC26 and EORTC QLQ‐C30 from baseline until the 2‐year follow up visit. 2. Sexual Drive, Function and Satisfaction

Another objective is to assess changes in sexual drive, function and overall satisfaction before and after treatment(s). This will be evaluated by tracking changes in domain scale scores and single‐item scores in the Brief Male Sexual Function Inventory (BMSFI), QLQ‐TC26 and supplementary anejaculation‐related questions, with assessments conducted from baseline to the 2‐year follow‐up visit.3. Progression‐Free Survival (PFS) and Overall Survival (OS)

The study also aims to evaluate PFS and OS rates to ensure they align with standard‐of‐care treatment outcomes.

The study seeks to assess the safety and complications associated with all treatments.

## 4 ELIGIBILITY CRITERIA

### 4.1 Inclusion criteria

Each participant must meet all of the following inclusion criteria to be enrolled in either the two cohorts within the study:

1. Willing and able to provide written informed consent
2. Male sex
3. Age ≥ 16 years
4. Histologically confirmed seminoma or NSGCT (biopsy/orchidectomy)
5. International Germ Cell Cancer Collaborative Group (IGCCCG) good prognosis group
6. Clinical stage II (standard of care cross‐sectional imaging) at time of study entry
7. Ability to comply with the protocol, including but not limited to, completion of the patient‐reported outcome questionnaires
8. ECOG performance status 0–1

#### 4.1.1 rRPLND de‐escalation group specific inclusion criteria

Participants must meet the following additional inclusion criteria:

1. Histologically confirmed seminoma (biopsy/orchidectomy).
2. Stage IIA and <3 cm IIB with ipsilateral lymph node(s) within rRPLND template.
3. Negative or mildly elevated serum tumour markers, defined as:
  - AFP (alpha‐fetoprotein) within normal range or <20 ng/ml if stable on at least two serial measurements.
  - BhCG (human chorionic gonadotropin) < 50 mg/ml.
  - LDH (lactate dehydrogenase) < 1.5x upper limit normal.
4. Fit for surgery, defined as meeting all of the following criteria:
  - Body mass index (BMI) not expected to preclude surgery as assessed by the investigator.
  - Charlson comorbidity index ≤3.
  - No significant cardio‐pulmonary disease, or other uncontrolled intercurrent illness that would limit fitness for surgery in the opinion of the investigator.

#### 4.1.2 Carboplatin AUC10 de‐escalation group‐specific inclusion criteria

Participants must meet the following additional inclusion criteria:

1. Histologically confirmed seminoma (biopsy/orchidectomy).
2. Serum tumour markers, defined by IGCCCG good prognosis criteria:
  - AFP < 10 ng/ml and non‐rising on serial testing.
  - Any BhCG.
  - LDH < 2.5x ULN.
3. Glomerular filtration rate by EDTA clearance over 25 ml/min (a measured creatinine clearance using Cockcroft and Gault would be allowed if unable to perform EDTA clearance).
4. Participants must be sterile or agree to use adequate contraception during the period of therapy.

### 4.2 Exclusion criteria

A participant will not be eligible for inclusion in this study if any of the following criteria apply:

1. Previous chemotherapy or radiotherapy for the disease under study.
2. Previous or concurrent malignancy other than testicular cancer, unless treated with curative intent and with no known active disease present for ≥2 years before enrolment and felt to be at low risk for recurrence by the treating physician (for example: non‐melanoma skin cancer or lentigo maligna; breast ductal carcinoma in situ; prostatic intraepithelial neoplasia; urothelial papillary non‐invasive carcinoma or urothelial carcinoma in situ).
3. Any condition that, in the opinion of the investigator, would interfere with evaluation of study intervention or interpretation of participant safety or study results, such as medical comorbidities impacting QoL or medical conditions or other disorders that would affect adherence to study requirements.

## 5 METHODS

### 5.1 Intervention

Participants will receive SOC treatments for good prognosis stage II seminoma and NSGCT or de‐escalated treatments (primary rRPLND with or without adjuvant treatment or Carboplatin AUC10) for stage II seminoma. Participants will be allocated to one of the following two cohorts based on whether the relevant de‐escalated treatment is adopted as an institutional SOC:

- rRPLND cohort: Participants with ipsilateral stage IIA or <3 cm IIB seminoma with negative/low for tumour markers will be offered rRPLND followed by adjuvant treatment or surveillance based on post‐operative histology as per institutional SOC.
- Carboplatin AUC10 cohort: Participants with stage II seminoma will be offered Carboplatin AUC10 by 3 cycles.

**FIGURE 1:**
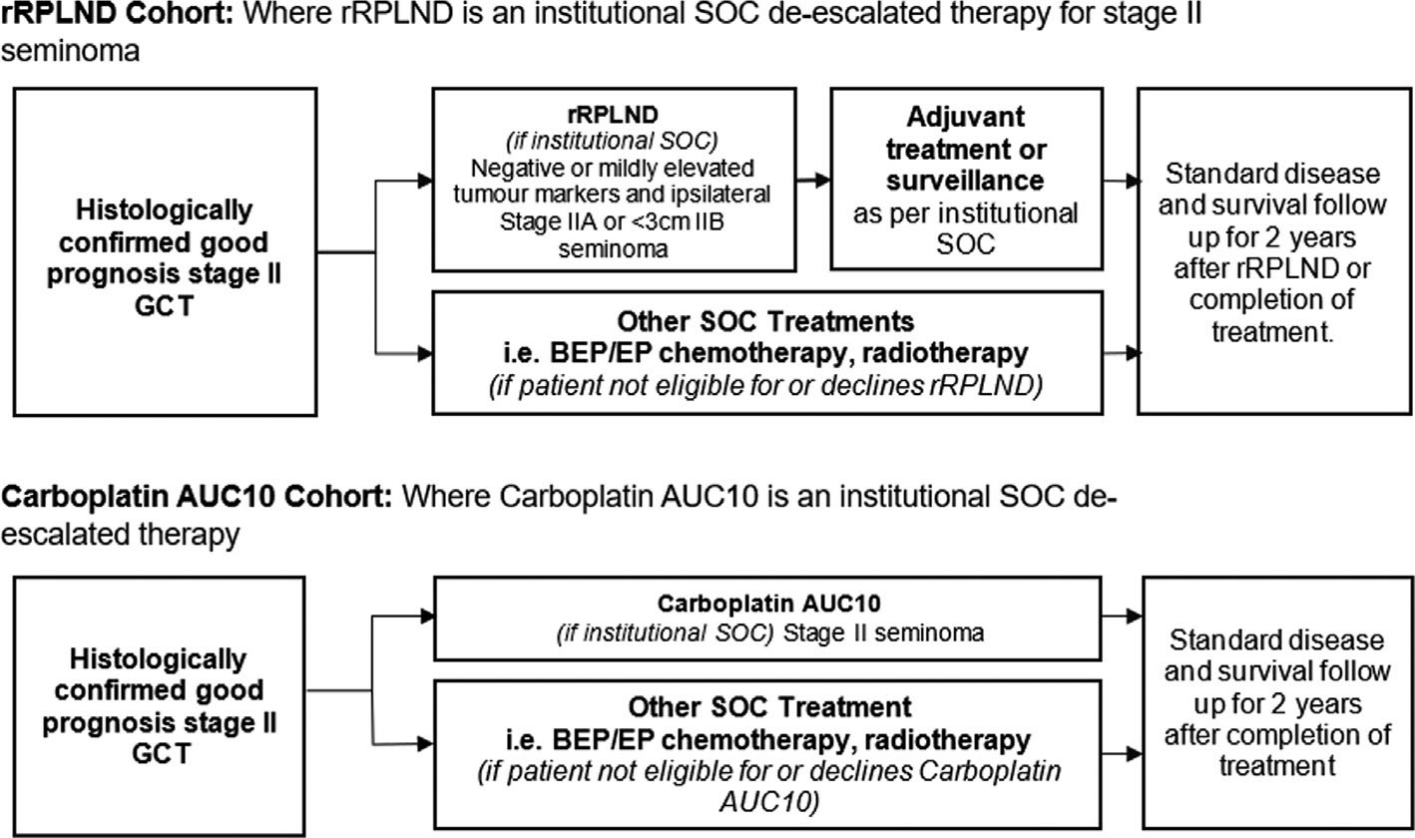
Flow diagram outlining the treatment cohort.

Participants deemed ineligible for Carboplatin AUC10 or rRPLND or those who decline this de‐escalation option will be offered either BEP or EP chemotherapy (seminoma and NSGCT) or radiotherapy with or without neoadjuvant Carboplatin AUC7 (seminoma) and will continue to be followed in the study. Chemotherapy treatment strategies and adjuvant treatments are left to the shared decision‐making between treating clinicians and participants and follow institutional SOC. The study flow diagram outlining the treatment cohorts is summarised in Figure 1.

#### 5.1.1 Institutional standard of care treatments

*rRPLND*: Participants undergoing rRPLND will have robot‐assisted laparoscopic surgery to remove ipsilateral retroperitoneal lymph nodes(s) as per local Trust procedures. For participants undergoing rRPLND, adjuvant treatment decisions are based on post‐operative histology and is left to the shared decision‐making between treating clinician and participants as per local SOC. The adjuvant treatments typically used following rRPLND are summarised in Table 1. Data will be collected on the type of adjuvant treatment delivered.

**TABLE 1:** Typically‐used adjuvant treatments following rRPLND.

Post‐operative nodal histology	Adjuvant treatment
pN0 or pN1 teratoma only	No adjuvant treatment
pN1 mixed/NSGCT	1x Bleomycin, etoposide and platinum (BEP), OR 2x Etoposide and cisplatin (EP)
pN1 seminoma	1x Carboplatin AUC7 or 10
pN1 positive surgical margins	2x BEP, OR 2x EP (NSGST), OR 2x Carboplatin AUC10 (Seminoma)

*Carboplatin AUC10*: Carboplatin AUC10 is typically given intravenously once every 21 days for 3 cycles (as per local Trust procedures).

*BEP/EP*: BEP is given intravenously every 21 days over 3 cycles while EP is given intravenously every 21 days over 4 cycles (as per local Trust procedures).

*Radiotherapy:* Radiotherapy is delivered with 30Gy in 15 fractions to para‐aortic nodes and 5Gy in 3 fractions to ipsilateral iliac nodes. Alternatively, a single cycle of Carboplatin (AUC7) is followed by 30Gy in 15 fractions to para‐aortic nodes (as per local Trust procedures).

All agents listed above are non‐investigational medicinal products licensed in the UK for treatment of TGCT. Information regarding anti‐cancer treatments will be collected for all participants throughout the follow‐up period.

### 5.2 Sample size

This is a 30‐participant feasibility study across 2 cohorts with an 18‐month recruitment period. Hence, sample size calculation is not appropriate, but the recruitment target is typical for feasibility studies.^13^

### 5.3 Study timeline

The tables below describe the participant timeline through the trial. Participants will attend visits according to the schedule outlined in Table 2 for the rRPLND cohort and Table 3 for the Carboplatin AUC10 cohort. These visits will include screening, treatment and follow‐up assessments to monitor progress and outcomes.

**TABLE 2:** Schedule of assessments for rRPLND cohort.

	Screening	rRPLND	2 weeks	6 weeks	12 weeks	6 months	9 months	12 months	15 months	18 months	21 months	24 months	Disease recurrence
	<6 weeks pre‐surgery		± 2 weeks	± 2 weeks	± 2 weeks	± 2 weeks	± 2 weeks	± 4 weeks	± 4 weeks	± 4 weeks	± 4 weeks	± 4 weeks	+4 weeks
**Informed consent**	X												
**Demographic data & medical history**	X												
**Blood tests** ^**1**^	X		X	X^7^	X	X	X	X	X	X	X	X	X
**EDTA creatinine clearance**				X^2^									
**Fitness for surgery**	X												
**Imaging (RECIST v1.1)**	X				X			X				X	X
**Institutional SOC treatment**		Participants will receive either rRPLND or other institutional SOC treatment if not eligible/decline rPLND Treatment details will be collected on the Case Report Forms (CRF)		Participants to receive adjuvant chemotherapy if applicable									
**Surgical outcome & complications**			X	X	X	X							
**Adverse events**		AEs will be collected from the date of consent until 6 weeks post rRPLND or the end of treatment depending on the allocated treatment.^6^											
**Tumour sample**	X^3^	X											
**Research blood sample**	X		X		X^7^								X
**Patient reported outcomes** ^**4**^	X		X	X	X	X		X		X		X	X
**Disease, treatment, survival FU** ^**5**^						X	X	X	X	X	X	X	X

^1^ Serum tumour markers, urea and electrolytes, liver function tests and full blood count. Tests may be carried out up to ≤72 hours before the day of treatment.

^2^ To be carried out prior to adjuvant treatment only for participants who undergo chemotherapy.

^3^ Archival FFPE tumour sample from previous biopsy or orchidectomy.

^4^ Patient reported outcome information collected via questionnaires: EORTC QLQ‐TC26, EORTC QLQ‐C30, BMSFI and supplementary questions.

^5^ Disease, treatment and survival follow‐up information may be collected in clinic or via a telephone call with the participant.

^6^ For participants that are not eligible/decline rRPLND, AEs will be collected from the date of consent until 6 months post the end of treatment depending on the allocated treatment.

^7^ Only for participants that receive adjuvant chemotherapy.

**TABLE 3:** Schedule of assessments carboplatin AUC10 cohort.

	Screening	Chemotherapy (cycle 1–3)	2 weeks	6 weeks	12 weeks	6 months	9 months	12 months	15 months	18 months	21 months	24 months	Disease recurrence
	<2 weeks prechemotherapy		± 2 weeks	± 1 week	± 2 weeks	± 2 weeks	± 2 weeks	± 4 weeks	± 4 weeks	± 4 weeks	± 4 weeks	± 4 weeks	+4 weeks
**Informed consent**	X												
**Demographic data & medical history**	X												
**Blood tests** ^**1**^	X	X	X		X	X	X	X	X	X	X	X	X
**EDTA creatinine clearance**	X												
**Imaging** *(RECIST v1.1)*	X		X					X				X	X
**Institutional SOC Treatment**		Participants will receive either Carboplatin AUC10 or other institutional SOC treatment if not eligible/decline Carboplatin AUC10. Treatment details will be collected on the CRF.											
**Adverse events**	AEs will be collected from the date of consent until 6 months post the end of treatment depending on the allocated treatment.												
**Tumour sample**	X^2^												
**Research blood sample**	X		X										X
**Patient reported outcomes** ^**3**^	X		X	X	X	X		X		X		X	X
**Disease, treatment and survival FU** ^**4**^						X	X	X	X	X	X	X	X

^1^ Serum tumour markers, urea and electrolytes, liver function tests and full blood count. Tests may be carried out up to ≤72 hours before the day of treatment. Test might be carried pre‐treatment for every cycle.

^2^ Archival FFPE tumour sample from previous biopsy or orchidectomy.

^3^ Patient reported outcome information collected via questionnaires: EORTC QLQ‐TC26, EORTC QLQ‐C30, BMSFI and supplementary questions.

^4^ Disease, treatment and survival follow‐up information may be collected in clinic or via a telephone call with the participant.

### 5.4 Methods of data collection

*Demographic data*: Age, sex, date of birth and race/ethnicity will be collected with participants' consent, along with a comprehensive medical history including previous conditions, surgeries, cancer history and detailed testicular cancer diagnosis.

*Blood samples*: Serum tumour markers (AFP, BhCG, LDH) will be taken at screening and analysed locally. Results will be recorded in CRF, with clinically significant treatment‐related abnormalities noted as AEs. For participants undergoing rRPLND, surgical outcomes will be recorded, including complications, hospital stay and recovery time, up to six months post‐surgery.

*Imaging*: Centres that perform rRPLND typically undertake a baseline PET scan prior to surgery to determine eligibility. Positron Emission Tomography (PET) is typically not used in centres treating participants with Carboplatin AUC10. PET is not mandated for this trial; however, if a baseline PET is undertaken as part of institutional SOC, the results will be collected. All tumour assessments will be performed using Computed Tomography (CT) scans following the schedule of assessments. If contrast CT is contraindicated, Magnetic Resonance Imaging (MRI) scans and non‐contrast CT scans may be used. RECIST v1.1 criteria will be used to assess participants response.

*Health‐related quality of life*: Will be assessed using standardised validated questionnaires (EORTC QLQ‐TC26, EORTC QLQ‐C30, BMSFI and additional bespoke enquires on anejaculation), self‐administered using paper‐based format by participants. Site staff will assist if needed and document reasons for any uncompleted questionnaires. Completed patient‐reported outcomes (PRO) questionnaires will be sent to the coordinating team, with originals kept in patient records.

*Adverse event*: Will be tracked from consent to six weeks post‐rRPLND and six months post‐chemotherapy. Unresolved AEs at study end will be followed up by the Investigator but without further recording in the CRF.

### 5.5 Data management

Data are captured using an electronic CRF (eCRF) designed with ORACLE Database 11 g. The system ensures secure data handling according to strict standard operating procedures (SOPs). Trained site personnel enter data directly into the eCRF, including serious AEs (SAEs), serious adverse reactions (SARs) and suspected unexpected serious adverse reactions (SUSARs), which are also recorded in a separate Pharmacovigilance (PV) database. Monitoring visits and results are documented in a Monitoring Database. Data storage and backups follow strict SOPs, with central management ensuring data validation, medical coding and query resolution. Final database closure involves comprehensive checks and approvals to ensure data integrity.

### 5.6 Data analysis

Summary statistics of overall participant retention within the trial until the 2‐year follow‐up visit will be presented. Full details of the analysis of all endpoints in the statistical analysis plan (SAP) will be provided, which will be finalised prior to any review or analysis of data.

*Analysis of participant populations*: The full analysis set (FAS) population and the safety set population will include all participants enrolled in the trial who received study treatment. Efficacy and safety analyses will be performed on these populations, respectively.

*HRQOL*: Summary statistics will be presented for items of each questionnaire by time point. Methods for scoring will follow the guidelines provided in the respective manuals. Further details on comparisons of questionnaires between time points and between arms will be provided in the SAP.

*PFS and OS*: The Kaplan–Meier (K‐M) methodology will be used to estimate the PFS and OS rates at 2 years, as well as the median PFS and OS for each treatment arm and for all participants. These estimates will be accompanied by 95% confidence intervals (CIs), and the K‐M curves will be plotted. The treatment effect will be evaluated by calculating the hazard ratio (HR) along with its corresponding CIs and p‐value, obtained from the Cox proportional hazards model.

### 5.7 Adverse event reporting and harms

The Safety Reporting Instructions for the THERATEST trial outline the procedures for reporting and managing AEs and SAEs. An AE is defined as any unfavourable medical occurrence in a participant, regardless of its relationship to the research procedure. SAEs are more severe, meeting specific criteria such as being life‐threatening or requiring hospitalisation.

AEs are recorded from the time of informed consent until the safety visit and are tracked in the eCRF. Certain events, such as elective hospitalisations, are excluded from SAE reporting. SAEs must be reported to the THERATEST Coordinating Team within 24 hours using the THERATEST SAE Reporting Form. The form must be fully completed, including details like event severity, relatedness to the study and outcome. The coordinating team verifies the form's completeness and liaises with the site for any additional information. If an SAE is deemed both related to the study and unexpected, a non‐CTIMP safety report must be sent to the Research Ethics Committee (REC) within 15 days. Follow up reports are required as new information arises, ensuring thorough documentation and communication with the sponsor.

## 6 PROTOCOL AMENDMENT

The trial manager disseminates protocol amendments to relevant parties. All versions have received approval from the sponsor, funding bodies and the ethics committee. The current version 5.0 is considered final with no substantive changes planned.

## 7 CONSENT

Informed consent will be obtained from participants before enrolling in the trial, and the Principal Investigator (PI) is responsible for this process at each site, ensuring that those delegated to obtain consent are authorised, trained and competent. The informed consent form for Carboplatin AUC10 cohort is included in Supplementary File 1, and for rRPLND cohort in Supplementary File 2.

## 8 TREATMENT AND SURVIVAL FOLLOW‐UP

All participants will be followed for treatment, survival and disease status information unless the participants request to be withdrawn from follow‐up or the study is terminated by the sponsor. Follow‐up information will be collected via telephone calls or review of patient medical records following the schedule of assessments.

## 9 MONITORING

A Trial Monitoring Plan will be developed and agreed by the sponsor and chief investigator (CI) based on the sponsor's risk assessment. Monitoring will consist of combination of activities performed by the sponsor, study team and trial committee members. The sponsor can audit any part of the study, including sites and central facilities and the funder may inspect the study where applicable. Sites must inform the sponsor if notified of any audit or inspection. The final locked dataset will only be accessible to the coordinating team, Study Statistician and CI. The final study dataset will also be available for review by the Trial Management Group (TMG) as part of their study oversight responsibilities.

## 10 PUBLIC AND PATIENT INVOLVEMENT (PPI)

The study concept and design have been thoroughly discussed with the coordinating team PPI group. We sought PPI feedback and incorporated their suggestions into the trial. The PPI group will also be engaged if any substantial amendments are planned that could affect patient participation in the THERATEST study.

## 11 CONFIDENTIALITY

Each participant will receive a unique identifiers number at consent. No participant will be identifiable in publications. Confidentiality will be maintained per the Data Protection Act (2018), the Research Governance Framework for Health and Social Care and Research Ethics Committee approval. All study data will be stored in line with the Medicines for Human Use (Clinical Trials) Regulations 2004 and the Data Protection Act.

## 12 DISCUSSION

There is a need for prospective trials to assess de‐escalation strategies for stage II seminoma, particularly to determine the efficacy, safety and toxicity of treatments. The lack of randomised trials in this area has led to varied institutional SOC, shaped by individual clinical experiences, making it difficult to reach an international consensus on treatment strategies.

THERATEST is the first study to prospectively evaluate standard of care treatments (multi‐cycle combination chemotherapy or external beam radiotherapy) and novel de‐escalation strategies (Carboplatin AUC10 and rRPLND with or without adjuvant chemotherapy) for good prognosis GCT. This study will contribute valuable insights to the literature by combining qualitative and quantitative data to comprehensively assess outcomes. The study will not only enhance our understanding of clinical efficacy but also provide a deeper insight into the patient‐reported outcomes, which is crucial for optimising treatment decisions within a shared decision‐making framework.

However, the study has several limitations. The relatively short follow‐up period of 2 years may be insufficient to fully assess long‐term disease relapse and treatment‐related effects. Additionally, the generalisability of the findings may be limited, as all rRPLND procedures will be performed by a small team of surgeons at a single centre. Furthermore, the lack of randomisation in treatment allocation restricts the study's ability to evaluate the feasibility of incorporating randomisation in future trials.

If successful in achieving its primary outcomes of recruitment and retention, THERATEST will lay the groundwork for a larger‐scale multicentre study, with the potential to inform broader evidence and shape future clinical guidelines. The study's findings will be crucial in addressing existing gaps in evidence and shaping the direction of future research on de‐escalation strategies for this rare cancer.

## AUTHOR CONTRIBUTIONS

CA, BT, CC, EL, CR, DN, RH, AR, JS and PR contributed to the research idea and study design. KN, CA, BT, CC, EL, CA, RG, TJ, CR, EM, DN, WC, RH, AR, JS, NAA and PR contributed to the development of the study protocol. NAA and PR led the development of the manuscript, and all other authors contributed to and approved the final document.

## CONFLICT OF INTEREST STATEMENT

CC has received honoraria from AstraZeneca, Janssen (J&J) and Bristol Myers Squibb and travel support from Merck. EL has received honoraria from BMS, MSD and Eisai and travel support from Pfizer, BMS and MSD. KN has received honoraria from Union Chimique Belge (UCB), Pfizer, AstraZeneca, Janssen and GSK/Tesaro and travel support from GSK/Tesaro. All other authors declare no conflicts of interest.

## ETHICS

Ethical approval has been granted (UK HRA REC reference: 23/LO/0972) protocol v5.0 dated 18 June 2025.

## Supporting information


**Data S1.** Supporting Information.

**Data S2.** Supporting Information.

**Data S3.** Supporting Information.
