# Supplementary material for: Therapy de‐escalation for testicular cancer (THERATEST): A multi‐centre observational cohort feasibility study of de‐escalation therapies for good prognosis stage II germ cell tumours
Source: BJUI Compass. 2025 Jul 29;6(8):e70057. doi: 10.1002/bco2.70057 (PMC12307540; doi:10.1002/bco2.70057)
Supplement: Supplementary file 1 — Data S1. Supporting Information. [file BCO2-6-e70057-s003.docx]

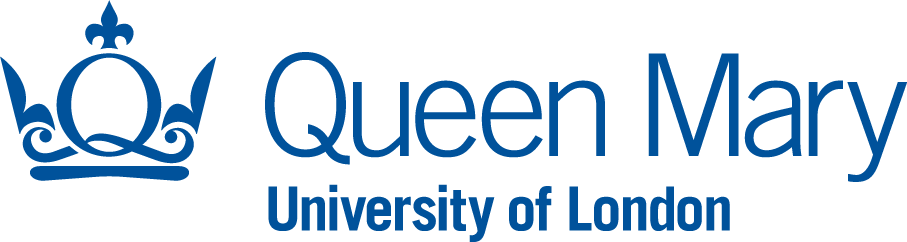


| **Title of Study:** | THERATEST: THERApy de-escalation for TESTicular cancer | | |
| --- | --- | --- | --- |
| **Cohort:** | Carboplatin AUC10 De-escalation Group or other institutional SOC treatment | | |
| **IRAS ID:** | 305109 | **REC Ref** | 23/LO/0972 |
| **Study Sponsor:** | Queen Mary University of London | | |
| **Site Name:** |  | **PI Name:** |  |

**PARTICIPANT INFORMATION SHEET**

We would like to invite you to take part in a research study. Before you decide whether to take part, it is important for you to understand why the research is being done and what it will involve. Please take time to read the following information carefully. After reading this form, you will have a chance to discuss the study with your family and friends. Ask us if there is anything that is not clear or if you would like more information. Take time to decide whether or not you wish to take part. If you decide to participate in the study, you will be asked to sign the Informed Consent Form. No study testing or study evaluations will be done before you have signed this consent form.

Contents

[1 What is the purpose of the study? 3](#_Toc155081232)

[2 How does the treatment work? 3](#_Toc155081233)

[3 Why have I been chosen? 4](#_Toc155081234)

[4 Do I have to take part? 4](#_Toc155081235)

[5 What are the alternatives for treatment? 4](#_Toc155081236)

[6 What will happen to me if I take part? 4](#_Toc155081237)

[7 What will happen to any samples I give? 7](#_Toc155081238)

[8 What do I have to do? 7](#_Toc155081239)

[9 Will my taking part in this study be kept confidential? 8](#_Toc155081240)

[10 Expenses and payments 9](#_Toc155081241)

[11 What are the possible benefits of taking part? 9](#_Toc155081242)

[12 What are the possible disadvantages and risks of taking part? 9](#_Toc155081243)

[13 Can I take other medication whilst on the study? 11](#_Toc155081244)

[14 What happens when the research study stops? 12](#_Toc155081245)

[15 What happens if there is a problem? 12](#_Toc155081246)

[16 What if relevant new information becomes available? 12](#_Toc155081247)

[17 What will happen if I don’t want to carry on with the study? 12](#_Toc155081248)

[18 Who is organising and funding the research? 12](#_Toc155081249)

[19 Who has reviewed the research? 13](#_Toc155081250)

[20 Who can I contact for further information? 13](#_Toc155081251)

[21 Indemnity/ Insurance 13](#_Toc155081252)

# What is the purpose of the study?

Testicular cancer is a type of cancer that develops in the testis. It is the most common cancer in young adult men. The seminoma type of testicular cancer make up just over half of all cases, and the remainder are non-seminomas. In stage II testicular cancer, cancer cells are suspected to have spread beyond the testicle to lymph nodes (glands) in the tummy (abdomen) or pelvis (or in rare cases can arise in the lymph nodes without the testis being involved). This type of cancer is usually first treated by surgery to remove the affected testis (orchidectomy). After surgery patients are normally given chemotherapy with multiple drugs or radiotherapy to the lymph nodes in the abdomen, or a combination of the two to reduce the risk of the cancer coming back (recurrence). These standard of care treatments have excellent cure rates, but they can cause some serious side effects which can affect a patient’s quality of life.

Typically, treating physicians decide on the appropriate treatment strategy based on international standards, and include scans and blood tests, as well as the type(s) of treatment available in their institutions. In some hospitals in the United Kingdom, physicians have been using alternative treatment strategies that cause less side-effects but have similar cure rates to standard of care for stage II seminoma. These novel strategies are called de-escalation treatments, and follow a principle that the same result can be achieved by giving less of a particular treatment for a disease with a high cure rate. These de-escalation treatments are chemotherapy with a single less toxic drug (Carboplatin AUC10) or keyhole surgery by robotic Retroperitoneal Lymph Node Dissection (rRPLND) to remove the lymph nodes with or without a low dose chemotherapy (Carboplatin AUC7) given afterwards.

There is no single widely adopted de-escalation treatment for stage II seminoma in the UK nor accepted international standards for these approaches. There are currently no studies that compare these types of treatment and their impact on patients’ cancers and quality of life. Hence, many patients could be missing out on an opportunity to reduce the side effects of treatment without compromising cure rates.

THERATEST is looking to collect data from 30 patients actively receiving de-escalation treatments or other standard of care treatments for stage II seminoma and non-seminoma in two UK hospitals. THERATEST is a feasibility study to determine whether patients are willing to be recruited, the impact of de-escalation treatments on patients’ cancers and quality of life, whether we should proceed with these treatments in a larger study, and if so how the study should be conducted. A feasibility study prepares the ground for a larger study and improves the chances of the subsequent study producing valuable evidence, and helps to avoid wasting precious resources on larger trials that are unlikely to be informative. We hope that information from THERATEST will bridge the current knowledge gap and allow clinicians to design bigger trials to actively compare the different treatment strategies.

# How does the treatment work?

The de-escalation treatment you will be offered in this study is the same treatment that you would receive as standard of care, based on your type of cancer and treatments available at your local hospital. Your hospital offers Carboplatin AUC10 as a de-escalation treatment for stage II seminoma. Carboplatin is a type of chemotherapy and is approved for the treatment of early testicular cancer. It kills cancer cells by interfering with their genetic material (DNA) and stopping them from multiplying. Carboplatin is given directly in the blood stream via a drip or via a central line once every 21 days for 3 cycles.

If you do not wish to receive Carboplatin AUC10 or your doctor feels this is not the best treatment for you, they may offer you other types of combination chemotherapy either bleomycin, etoposide and platinum (BEP) or etoposide and platinum (EP) chemotherapy (seminoma or non-seminoma) or radiotherapy with or without chemotherapy given before treatment (seminoma). All these are also standard of care treatments, but are not de-escalation treatments. The decision on which treatment is best for you will be based on discussions between you and your doctor and will be in line with your local hospital practices. Your doctor will give you more information on these treatments during your discussions.

# Why have I been chosen?

You have been invited to take part in this study because you have stage II seminoma or non-seminoma and your local hospital offers Carboplatin AUC10 as a de-escalation option. All treatment decisions and the majority of assessments will be the same as standard of care. Your doctor will discuss this with you in further detail.

If you are not eligible to receive Carboplatin AUC10 or if you do not wish to, your doctor may offer you other standard of care treatments for your cancer such as combinations of chemotherapy drugs or radiotherapy. In this case, we will still collect data of the treatments you are receiving and how well they control your cancer, and the impact on your quality of life.

# Do I have to take part?

No, it is up to you to decide whether or not to take part. If you choose to take part, you will be asked to sign an informed consent form and you will be given a copy of this document to keep. If you do not wish to take part in the study you do not have to give a reason. You will not be disadvantaged in any way, and it will not affect the standard of care you receive. This also applies if you initially decide to take part and then change your mind at a later date.

# What are the alternatives for treatment?

If you decide not to participate you will follow the standard care pathway for this type of cancer, which is the same as the treatment you would have received in this study. Talk to your doctor about your choices before you decide whether to take part in this study.

# What will happen to me if I take part?

After your doctor has answered all of your questions about the study and you have given written consent by signing this form, several examinations and laboratory tests will be done to make sure you are eligible to receive these treatments. This is called the screening period. All of these tests are the same as those you would have as part of standard of care. If you had some of these to diagnose your cancer, they may not need to be repeated. The decision will be made by your study doctor.

If your doctors confirm you are eligible to participate in this study, you will receive one of two possible treatment strategies:

- De-escalation treatment with Carboplatin AUC 10 at 21-day intervals. Each 21-day interval is called a cycle. Carboplatin is administered by injecting into a vein through a cannula over approximately 60 minutes in hospital. You will receive three cycles of this treatment.

*OR*

- Other types of standard of care treatments such as combination chemotherapy (e.g. bleomycin, etoposide and platinum (BEP) or etoposide and platinum (EP)) or radiotherapy. These will be offered to you if you do not wish to receive Carboplatin AUC10 or if your doctor believes this is not the best treatment option for you. The details of the duration and frequency of these treatments will be given to you by your doctor.

When you complete your treatment we will follow your progress for 2 years. This will allow us to collect data on long-term side effects, how well the treatment controlled the growth of your cancer as well as the impact it had on your quality of life. All visits will be in line with standard of care visits and you won’t have to attend additional hospital visits.

Each treatment has a slightly different schedule of visits and assessments. Below you will find a table summarising the assessments you will have in this study. Those highlighted are additional to standard of care. If you have questions about the schedule of assessments below, please ask your medical team and they will be happy to explain further.

**Table 1: THERATEST Schedule of Assessments**

|  |  |  | **Weeks** | | | **Months** | | | | | | |  |
| --- | --- | --- | --- | --- | --- | --- | --- | --- | --- | --- | --- | --- | --- |
| **Visit 🡪** | **Screening** | **Chemotherapy** | **2** | **6** | **12** | **6** | **9** | **12** | **15** | **18** | **21** | **24** | **Disease recurrence** |
| **Window 🡪** | **-14 to 0 days** | **Cycle 1-3** | **± 2 wk** | | | **± 2 wks** | | **± 4 wks** | | | | | **+4 wks** |
| **Consent to the study** | X |  |  |  |  |  |  |  |  |  |  |  |  |
| **Demographic data** *(date of birth, sex, race)* | X |  |  |  |  |  |  |  |  |  |  |  |  |
| **Medical history** | X |  |  |  |  |  |  |  |  |  |  |  |  |
| **Blood test**  *(cancer markers, haematology and biochemistry)* | X | X | X |  | X | X | X | X | X | X | X | X | X |
| **EDTA Creatinine Clearance** | X |  |  |  |  |  |  |  |  |  |  |  |  |
| **Scans of your chest, abdomen and pelvis** (to assess your cancer) | X |  | X |  |  |  |  | X |  |  |  | X | X |
| **Institutional standard of care Treatment** |  | You will receive either Carboplatin AUC10 or other institutional standard of care treatment if you are not eligible or decline Carboplatin AUC10. | | | | | | | | | | |  |
| **Review of illnesses / symptoms** | Illnesses and symptoms will be collected from the date you give consent until 6 months after the end of your treatment. | | | | | | | | | | | |  |
| **Tumour sample** | X^[[1]](#footnote-1)^ |  |  |  |  |  |  |  |  |  |  |  |  |
| **Research blood sample ^[[2]](#footnote-2)^** | X |  | X |  |  |  |  |  |  |  |  |  | X |
| **Quality of life questionnaire ^[[3]](#footnote-3)^** | X |  | X | X | X | X |  | X |  | X |  | X | X |
| **Follow up** |  |  |  |  |  | X | X | X | X | X | X | X | X |

# What will happen to any samples I give?

When consenting to take part in this study, you also have the option of consenting to provide the required blood and tissue samples so that future research can be performed. This party of the study is optional. This will allow us to learn more about your cancer, and try to see what effect the study drugs have on your cancer’s biology.

The samples and de-identified pathology reports (all personal details will be removed) will be sent to the coordinating centre in the UK, at the following address: Queen Mary University of London, Charterhouse Square, London, EC1M 6BQ.

All collected samples will only be used for future ethically approved research. Samples will be stored in a licensed tissue bank to be used for future ethically approved studies, which may include genetic and molecular testing on the samples. Samples will be stored under the Barts and the London Queen Mary’s School of Medicine and Dentistry Tissue Bank (HTA Licensing number: 12199). It may be used by Queen Mary University of London for ethically approved research within a hospital, university, non-profit institution or a company laboratory within/outside the UK or EU. Samples will be lawfully disposed of, when necessary. A minimal de-identified dataset with information around your type of disease, treatment received and response to treatment will be shared with the Tissue Bank. This will allow researchers to interpret the results of their analysis. A copy of this consent form will be shared with the tissue bank in accordance with Human Tissue Authority standards to demonstrate your consent to your samples being used for future research, but this will not be shared with individual researchers who will have access to your samples and data.

Information from any research will be from all the study participants as a group, not just from your samples. You will not be informed of new analyses on the samples as these are preliminary tests, the meaning of which is unclear and are not expected to influence your care. The test results will not be given to any insurance company, your employer, your family, your GP/family doctor, or any other doctor who treats you now or in the future.

If you decide to leave the study at any point, we would like to be able to keep any samples and data collected up to that point for analysis. It is your right however, to request that all samples and data collected prior to you leaving the study are destroyed. If these are your wishes, please make this clear to your treating physician so that they can document this in your medical notes. In all other instances, the samples collected up to that point will still be analysed as part of the study and be retained for future analysis.

# What do I have to do?

If you become a participant in the study, you will be required to follow the study procedures. This means that you should attend all scheduled study visits and take the study medication. If you decide to take part in this study, you should not be involved in any other drug trial at the same time. You should inform the treating physician of any other medications you are taking or new medication that is prescribed for you in order to establish whether it is safe for you to continue this whilst taking the study medication. If you are admitted to any hospital you should inform them that you are on a study and give your study doctor’s contact details.

# Will my taking part in this study be kept confidential?

Yes. All information which is collected about you during the course of the research will be kept strictly confidential. If you consent to take part in the research, you will be given a unique trial number that will be used to identify you throughout the study along with your date of birth. The people conducting the study will abide by the General Data Protection Regulation (GDPR) and Data Protection Act 2018 and the rights you have under this Act.

Queen Mary University of London (Queen Mary) is the Sponsor for this study and it is based in the United Kingdom. Queen Mary will use information from you and/or your medical records in order to undertake this study and will act as the data controller for this study. This information will include your date of birth and health information, which is regarded as a special category of information.

People who do not need to know who you are will not be able to see your name or contact details We will keep all information about you safe and secure.  Once we have finished the study, we will keep some of the data so we can check the results. We will write our reports in a way that no-one can work out that you took part in the study. Your hospital will keep identifiable information about you from this study for 25 years after the study has finished.

If you agree to take part in this study, you will have the option to take part in future research using your data saved from this study. Any leftover tissue along with a minimal de-identified dataset will be transferred to a Tissue Bank to allow future research in this disease area (See Section 7 for more details). A copy of this consent form, which includes your full name, will be shared with the tissue bank in accordance with Human Tissue Authority standards to demonstrate your consent to your samples being used for future research.

**What are your choices about how your information is used?**

- You can stop being part of the study at any time, without giving a reason, but we will keep information about you that we already have.
- If you choose to stop taking part in the study, we would like to continue collecting information about your health from central NHS records. If you do not want this to happen, tell us and we will stop.
- We need to manage your records in specific ways for the research to be reliable. This means that we won’t be able to let you see or change the data we hold about you.
- If you agree to take part in this study, you will have the option to take part in future research using your data saved from this study.

**Where can you find out more about how your information is used?**

You can find out more about how we use your information:

- at <http://www.jrmo.org.uk/performing-research/conducting-research-in-the-nhs/setting-up-a-study/>
- at www.hra.nhs.uk/information-about-patients/
- on the leaflet available from [www.hra.nhs.uk/patientdataandresearch](http://www.hra.nhs.uk/patientdataandresearch)
- by asking one of the research team
- by sending an email to [data-protection@qmul.ac.uk](mailto:data-protection@qmul.ac.uk)

If you wish to take part in this study, we will ask your permission to contact your GP to let them know of your potential participation in the study. This is done so that all the doctors involved in your care are aware of the medicines you are taking. They can also tell us if there are any medical reasons why you should not take part in the study. Your hospital notes will also state that you are in the study.

# Expenses and payments

You will not be paid for participating in this study. The number of visits you will be asked to attend is the same as those you would attend if you were not participating in the study. If you have to attend additional visits then reimbursement of reasonable travel costs (up to £35 for each visit) can be sought for these additional visits. Your doctor or research nurse will be able to provide you with more information on how to claim these expenses. You will need to collect receipts for any travel expenses you wish to claim. If you have future life, travel or private medical insurance you should check with the company before agreeing to take part in the trial whether participation is considered a ‘material fact’ that should be reported. You will need to do this to ensure that your participation will not affect your insurance.

# What are the possible benefits of taking part?

This trial aims to find out information that may help people with testicular cancer. The treatments you will receive are the same as standard of care treatments. There is no additional benefit over and above the standard of care.

# What are the possible disadvantages and risks of taking part?

Any treatment you may receive as part of this study will be standard of care at your local hospital. Participating in this study will not put you at any additional risk compared to standard of care.

There are risks, discomforts and inconveniences associated with any anti-cancer treatment. These vary from person to person and deserve careful thought. Everyone taking part in the study will be followed carefully for any side effects. However, doctors and the study Sponsor do not know all the side effects that may happen, and there may be unknown side effects that could occur. Side effects can vary from mild to very serious. Your doctors may give you drugs to help lessen side effects. Many side effects go away soon after you stop what is causing them. In some cases, side effects can be serious, long lasting, and/or may never go away. No one can predict whether you will have some, all, or none of these, or how severe they may be. It is important that you tell your study doctor or research nurse about any problems you have at each hospital visit. You can telephone either of them between visits if you are concerned and you will find their telephone numbers printed at the end of this information sheet.

**Chemotherapy Side Effects:**

- Carboplatin AUC10 chemotherapy
- BEP (Bleomycin, Etoposide, Cisplatin) or EP (Etoposide, Cisplatin) chemotherapy

The chemotherapy is administered directly into a vein through a cannula. You may experience some pain, redness, swelling or itching at the site where the drug is administrated. The side effects seen in people treated with chemotherapy vary from person to person. Not everyone will experience all the side effects, and their severity can differ. This guide explains what to expect:

| **Common risks (1 in 10 patients)** |
| --- |
| - Neutropenia - Thrombocytopenia - Anaemia - Nausea and Vomiting - Loss of Appetite and Weight Loss - Hair Loss - Diarrhoea or Constipation - Skin and Nail Changes - Tummy (Abdominal) Pain - Lung problems/ inflammation - Liver Issues - Weakness - Changes to Mineral Levels leading to muscle spasm - Sore Mouth |
| **Occasional Risks (Between 1 and 10 in 100 patients)** |
| - Allergic Reactions - Blood Clots - Heart Problems - Hearing change - Numbness and tingling in hands and feet |
| **Rare Risks ( Less than 1 in 100 patients)** |
| - Pain Where the Cancer Is - Confusion - Blistering - Vision problems - Serious kidney problems - Secondary cancer |

**Radiotherapy** side effects can include feeling tired and weak, sickness and diarrhoea. The skin in the treatment area can get red and sore (like a mild sunburn). Although testicles will be protected from radiotherapy beams, there is a small risk that your remaining testicle could get a dose of radiation. Your doctor will give instructions about fathering a child when having radiotherapy treatment.

As with any drug, unknown risks and side effects are also possible, and you could experience a side effect that has not been anticipated. There is also a chance that other medications you may be taking could interact with any anti-cancer treatment you are receiving. For your safety, you must tell the study doctor or research nurse about all medications you are taking before you start the study. Also, please tell the doctor or research nurse before starting any non-study medications while you are on the study, including any over the counter medicines such as cough and cold remedies.

For more information about risks and side effects, ask your study doctor. You should talk to your study doctor about any side effects that you have while taking part in the study. The study doctor will take steps to treat any side effects if they appear.

**Risks associated with taking blood:** Risks associated with drawing blood from your arm include pain, bruising, light-headedness and on rare occasions, infection.

**Risks of Exposure to Radiation and Contrast Material:** A CT scan is a special test used to study the internal organs of your body. If you take part in this study you will have 5 CT chest – abdomen – pelvis scans with contrast. If contrast is not indicated, you may have MRI scans or non-contrast CT scans.

The 5 CT chest-abdomen-pelvis are part of your routine care. If you take part in this study you will not undergo any additional scans. These procedures use ionising radiation to form images of your body and/or provide treatment and/or provide your doctor with other clinical information. Ionising radiation can cause cell damage that may, after many years or decades, turn cancerous. The chances of this happening to you are the same whether you take part in this study or not.

As part of a CT scan, contrast material may need to be taken by mouth and/or injected into your vein to make certain organs and tumour sites visible on the scan. Oral contrast may cause side effects such as nausea, constipation, diarrhoea, and abdominal bloating. Pain, bruising, redness, swelling, and/or infection may occur at the site where a needle is inserted to administer the contrast material into your vein. You may have an allergic reaction to the contrast material. Lastly, you may feel uncomfortable during the scan since you are not allowed to move during the procedure and may experience claustrophobia (fear of being in small places).

MRI scans use magnetic fields and radio waves to form images of your body. MRI scans are painless and safe and don’t expose the body to ionising radiation. You may feel uncomfortable during the scans since you are not allowed to move during the procedure and may experience claustrophobia.

**Possible risk to an unborn child:** Your doctor will give you specific advise depending on the type of treatment you receive. It is important to use highly effective methods of contraception while receiving anti-cancer treatment and for a certain time after completion of treatment. These drugs may harm a developing baby and your doctor will advise you not to get pregnant or make someone pregnant while having AUC10 treatment.

# Can I take other medication whilst on the study?

It is very important to tell your study doctor about all the medicines you take, including prescription and non-prescription medicines, vitamins, herbal supplements, and any new medicines you need to take. You need to know all the medicines you are taking. Keep a list of them with you to show your study doctor and pharmacist when you get a new medicine. You should not start or stop taking any medicine or supplement before you talk with the study doctor.

**Vaccinations**

Vaccinations can reduce your risk of getting certain infections. Your doctor or nurse may talk to

you about having vaccinations. Doctors usually recommend that people with cancer have a flu vaccination and a coronavirus (covid) vaccination. These are both inactivated vaccinations that can help reduce the risk of infection. People with weak immune systems can have these, as they are not live vaccinations.

If your immune system is weak, you need to avoid live vaccinations. This is because they can

make you unwell. Live vaccines contain a very weak version of the illness they are vaccinating

you against. Your cancer doctor or GP can tell you more about live and inactivated vaccinations.

# What happens when the research study stops?

All treatments received in this study are standard of care treatments and will be received in accordance with local hospital guidelines. Participating in this study does not affect the type or treatment you receive or how long you receive it for.

# What happens if there is a problem?

Queen Mary University of London has agreed that if you are harmed as a result of your participation in the study, you will be compensated provided that, on the balance of probabilities, an injury was caused as a direct result of the intervention or procedures you received during the course of the study. These special compensation arrangements apply where an injury is caused to you that would not have occurred if you were not in the trial. These arrangements do not affect your right to pursue a claim through legal action.

Please contact Patient Advisory Liaison Service (PALS)/Independent Advise Support Service (IASS - Scotland)/ XXX Health Board Patient Liaison Service (site to delete as appropriate) if you have any concerns regarding the care you have received, or as an initial point of contact if you have a complaint. Please telephone <<Insert PALS/IASS/Health Board Patient Liaison Service Telephone Number>> or email <<Insert PALS Email/IASS/Health Board Patient Liaison Service >> you can also visit PALS by asking at any hospital reception.

# What if relevant new information becomes available?

Sometimes during the course of a research project new information becomes available about the drugs that are being studied. If this happens your doctor will tell you about it and discuss whether you want to continue in the study. If you decide to withdraw, your doctor will make arrangements for your care to continue. If you decide to continue in this study you will be asked to sign an updated consent form.

# What will happen if I don’t want to carry on with the study?

You are free to withdraw from the study at any time and do not have to give a reason. Your future treatment will not be affected and your doctor will discuss this with you. We would like your permission to continue to receive information on your progress. If you choose to stop taking part in the study, we would like to continue collecting information about your health from your hospital. If you do not want this to happen, tell us and we will stop. We need to manage your records in specific ways for the research to be reliable. This means that we will not be able to let you see or change the data we hold about you. If you decide that we may have no further information from you for the study, we will need to use the data and samples collected up to the time of your withdrawal. This will include samples that you have given your consent for use in future research.

# Who is organising and funding the research?

This is an investigator-initiated study. Professor Prabhakar Rajan of Queen Mary University of London is the Chief Investigator. Queen Mary University of London is sponsoring the study. Barts Charity has agreed to fund the costs of the trial, but neither your doctors nor any other members of the clinical research teams will be paid for participating in this study, except for payments to cover study-related expenses.

# Who has reviewed the research?

This study has been through a peer review process. A peer review involves the examination of an author’s work by other experts in the same field. These referees each return an evaluation of the work which may include suggestions of improvements if necessary. Your local NHS Trust has been given approval for the study to take place at your hospital. The study has also been reviewed by the Riverside Research Ethics Committee (reference 23/LO/0972). This research has also been reviewed and approved by the Chief Investigator’s Institution.

# Who can I contact for further information?

For questions about the study you may contact your research doctor or nurse. The contact details are below: Dr <<Insert Doctor Name>> Telephone: <<Insert Doctor Telephone Number>> Or; Research Nurse <<Insert Research Nurse Name>> Telephone: <<Insert Research Nurse Telephone Number>>

**For information about your disease:** Cancer Research UK provides general information for patients about cancer and its treatment on their website, www.cancerresearchuk.org. The information can also be available over the phone by contacting a cancer information nurse on free-phone 0808 800 4040.

We are Macmillan provides support and counselling to help people living with cancer. Information can be found on their website, www.macmillan.org.uk, or by free-phone on 0808 808 0000.

# Indemnity/ Insurance

The insurance that Queen Mary University of London has in place provides cover for the design and management of the study as well as "No Fault Compensation" for participants, which provides an indemnity to participants for negligent and non-negligent harm.

**INFORMED CONSENT FORM**

| **Title of Study:** THERATEST: THERApy de-escalation for TESTicular cancer  **IRAS ID:** 305109  **THERATEST Screening Number** *(to be recorded after consent):* ***_________________*** | | | |
| --- | --- | --- | --- |
| **To be completed by participant:** | | | |
| **Please initial the box next to each statement to indicate agreement** | | | |
| 1. I confirm that I have read and understood this patient information sheet and have been given a copy to keep. I have had the opportunity to consider the information, ask questions and have had these answered satisfactorily.   PIS Version and Date: | | | Initials |
| 1. I understand that my participation is voluntary and that I am free to withdraw at any time, without giving any reason and without the standard of my medical care or legal rights being affected. | | | Initials |
| 1. I understand that relevant sections of any of my medical notes and data collected during the study may be looked at by responsible individuals from regulatory authorities and Sponsor representatives from Queen Mary University of London where it is relevant to my taking part in this research. Your individual NHS Trust may require access to these records to ensure that the trial is being conducted properly, in accordance with the data protection act. I give permission for these individuals to have access to my records. I understand both researchers and participants will be fully aware of the treatment regimen undertaken throughout the participation on the study as this is an open label clinical trial. | | | Initials |
| 1. I understand and agree that my GP/family doctor will be informed in writing of my decision to participate in the THERATEST study. | | | Initials |
| 1. I agree to take part in the above study. | | | Initials |
| **The below 3 points are OPTIONAL** | | | |
| 1. I agree to provide the required blood and tissue samples so that future research can be performed | | | Initials |
| 1. I understand that my blood and tissue samples will be stored for future ethically approved research. This research may involve national and international collaborations. I agree that samples and data collected from me may be stored at Queen Mary University of London and transferred within or outside the UK for further ethically approved studies, after being pseudo-anonymised for *future research*. I agree that a copy of my consent form can be shared with the Tissue Bank as evidence of my consent. | | | Initials |
| 1. I understand that analysis of blood and tissue samples may include genetic and molecular tests for *future research*. | | | Initials |
| **Confirmation of Consent** | | | |
|  |  |  | |
| **Name of Participant**  (BLOCK CAPITALS) | **Date** *(dd/mm/yyyy)* | **Signature** | |
|  |  |  | |
| **Name of Person Taking Consent**  (BLOCK CAPITALS) | **Date** *(dd/mm/yyyy)* | **Signature** | |

*(1 copy (original) for participant; 1 copy (original) for researcher (ISF); 1 copy to be kept in participant’s medical records; 1 copy for the tissue bank)*

1. The screening sample would have been collected as part of your normal clinical care and you do not have to undergo an additional biopsy. [↑](#footnote-ref-1)
2. This is additional to standard of care. Approximately 20ml of blood at each point indicated in the table above. This is approximately 1 – 2 tablespoons. [↑](#footnote-ref-2)
3. These questionnaires are additional to standard of care. They will help us understand that impact that cancer and anti-cancer treatments have on your day-to-day life. [↑](#footnote-ref-3)
