# Supplementary material for: Therapy de‐escalation for testicular cancer (THERATEST): A multi‐centre observational cohort feasibility study of de‐escalation therapies for good prognosis stage II germ cell tumours
Source: BJUI Compass. 2025 Jul 29;6(8):e70057. doi: 10.1002/bco2.70057 (PMC12307540; doi:10.1002/bco2.70057)
Supplement: Supplementary file 3 — Data S3. Supporting Information. [file BCO2-6-e70057-s001.docx]

**THERATEST Study Group**

**Chairman:** Professor Prabhakar Rajan (Queen Mary University of London)

**Urologists:** Walter Cazzaniga, David Nicol, and Erik Mayer (The Royal Marsden Hospital NHS Foundation Trust)

**Oncologists:** Nasreen Abdul Aziz, Kenrick Ng, and Jonathan Shamash (Barts Health NHS Trust), Constantine Alifrangis (University College London Hospitals NHS Foundation Trust), Ben Tran and Elizabeth Liow (Peter MacCallum Cancer Centre), Ciara Conduit (Royal Hobart Hospital), Robert Huddart and Alison Reid (The Royal Marsden Hospital NHS Foundation Trust)

**Clinical Trialists:** Clare Relton (Queen Mary University of London)

**Trial co-ordinators:** Charlotte Ackerman, Ramona Georgescu, and Tanim Jamal (Queen Mary University of London)
